# Simultaneous multiple post-labelling delay ASL MRI and [^18^F]FDG PET in a mixed memory clinic population and healthy controls

**DOI:** 10.1007/s00259-025-07736-8

**Published:** 2026-01-12

**Authors:** Otto M. Henriksen, Oriol P. Calvo, Frederik J. Bruun, Marie Bruun, Steen G. Hasselbalch, Kristian S. Frederiksen, Adam E. Hansen, Ian Law, Ulrich Lindberg

**Affiliations:** 1 Department of Clinical Physiology and Nuclear Medicine, Copenhagen University Hospital - Rigshospitalet, Copenhagen, Denmark; 2 Department of Nuclear Medicine, Zealand University Hospital, Køge, Denmark; 3 Department of Clinical Medicine, Faculty of Health and Medical Science, University of Copenhagen, Copenhagen, Denmark; 4 Department of Neurology, Danish Dementia Research Centre, Copenhagen University Hospital - Rigshospitalet, Copenhagen, Denmark; 5 Department of Radiology, Copenhagen University Hospital - Rigshospitalet, Copenhagen, Denmark

**Keywords:** PET/MRI, Arterial spin labelling, Positron emission tomography, [^18^F]fluoro-deoxy-glucose, Dementia

## Abstract

**Purpose:**

To assess the quantitative and visual concordance of multiple post-labelling delay (multi-PLD) arterial spin labelling (ASL) MRI cerebral blood flow (CBF) measurements and [^18^F]-fluoro-deoxyglucose (FDG) PET in a mixed memory clinic population.

**Methods:**

Hybrid [^18^F]FDG PET/MRI including multi-PLD pseudo continuous ASL from 96 memory clinic patients and 38 elderly controls were analysed along with ASL data from 12 healthy young volunteers. ASL image interpretability, concordance with [^18^F]FDG PET, and value of Z-score maps were rated visually. Regional associations of CBF with [^18^F]FDG uptake ratio (SUVr) were investigated by univariate regression and mixed linear models. Also influences of age, disease stage and vascular pathology on ASL interpretability and concordance, and whole cortex spatial coefficient of variation (sCOV) were analysed.

**Results:**

ASL CBF maps were non-comparable to [^18^F]FDG PET, i.e. uninterpretable or discordant, in 53% of patients, 37% of elderly controls, and 8% of young controls. Only 14% of patient ASL MRI scans were concordant with [^18^F]FDG PET. Z-score maps were mainly of value in partially concordant scans. Increasing sCOV was strongly associated both with disease severity and with decreasing ASL interpretability and concordance, and allowed for identification of uninterpretable scans with 95% sensitivity and 90% specificity. Whole cortex CBF and [^18^F]FDG SUVr values showed similar distribution across groups, but low to moderate regional associations.

**Conclusions:**

Multi-PLD ASL provided quantitative CBF measurements correlating with disease severity, but poor image quality and low regional concordance in head-to-head comparison with [^18^F]FDG PET imaging restricts the clinical use in memory clinic patients.

**Supplementary Information:**

The online version contains supplementary material available at 10.1007/s00259-025-07736-8.

## Introduction

In patients suspected of neurodegenerative dementia disorder, [^18^F]fluoro-deoxy-glucose (FDG) positron emission tomography (PET) is widely used to assess neuronal function and integrity, and is a valuable adjunct to structural imaging by computed tomography (CT) or magnetic resonance imaging (MRI). As opposed to pathology specific biomarkers, e.g. for amyloid accumulation, [^18^F]FDG PET is highly versatile by allowing identification of various disease specific patterns [1], also at an early disease stage [2], and can additionally be used for assessment of disease progression [3].

Arterial spin labelling (ASL) MRI allows assessment of regional cerebral blood flow (CBF) without use of contrast agent or radiation exposure, providing an attractive alternative to more costly and invasive functional brain imaging modalities such as [^15^O]H_2_O PET. Since its introduction more than three decades ago [4], ASL has been applied in numerous studies in basic, translational and clinical research, and recommendations for clinical use of ASL for various clinical indications including assessment for cognitive impairment due to neurodegenerative disorders have been published [5, 6]. Other studies have further demonstrated that ASL may quantify regional CBF and CBF reactivity with fair agreement with PET reference techniques in healthy volunteers [7, 8]. ASL can be included in standard MRI protocols and potentially reduce the need for (or altogether replace) [^18^F]FDG in dementia work-up, or in hybrid PET/MRI protocol combined with non-FDG PET tracers, e.g. for amyloid imaging, and, thus, allow for a more comprehensive diagnostic evaluation.

Several studies comparing ASL and [^18^F]FDG PET in dementia patients have been published. A recent meta-analysis concluded that ASL could show similar patterns as [^18^F]FDG PET at the group level, but also that ASL provided lower sensitivity than [^18^F]FDG PET both for detection and severity of regional abnormalities, and for diagnosis in well-defined disease states [9]. Limitations of comparative studies of ASL and [^18^F]FDG PET in dementia work-up include variations of ASL techniques applied, small samples typically including only well-defined diagnostic groups, and application of group analysis rather than single scan readings required for clinal diagnostic use.

A main limitation of ASL is inherently poor SNR, sensitivity to motion and variations in arterial transit time (ATT, time from labelling to arrival in tissue) compromising image quality and clinical usefulness. Prolonged ATT may be observed both within the brain typically leading to artefacts in the watershed areas, and between individuals leading to global artefacts with CBF maps dominated by vascular structures. Consequently, a single standard optimal post-labelling delay (PLD, i.e. time from labelling to readout) may not be possible. These issues may in particular apply to elderly subjects with vascular pathology, leading to increased inhomogeneity in the CBF map as reflected in a higher spatial coefficient of variation (sCOV) [10]. By acquiring data at multiple PLDs it may be possible to alleviate the challenges of ATT variability, and with subsequent modelling of multi-PLD data to calculate absolute CBF [11]. Such an approach has now been implemented in the current Alzheimer’s Disease Neuroimaging Initiative (ADNI-4) [12].

In a previous study we validated a 2D multi-PLD pseudo-continuous ASL (PCASL) sequence against the accepted reference method ([^15^O]H_2_O PET) in healthy subjects demonstrating the ability to quantitate CBF across a range of perfusions states [7]. Potentially, absolute quantification of CBF measurements could add information to [^18^F]FDG-PET by demonstrating globally impaired brain function that may not be recognized by semi-quantitative analysis of standard clinical [^18^F]FDG-PET. Studies investigating the potential of multi-PLD ASL in dementia are very sparse and to our knowledge no previous studies have compared such approaches against [^18^F]FDG PET.

The present study aimed to evaluate concordance between a validated multi-PLD PCASL technique and [^18^F]FDG PET in a mixed memory clinic population and in healthy controls simultaneously acquired on a hybrid PET/MR scanner. Specifically, we aimed to investigate image quality and factors that may influence image quality adversely, and similarity of regional abnormalities and correlation of quantitative CBF measurements with regional [^18^F]FDG distribution.

## Methods

### Participants

#### Healthy controls

A total of 44 healthy aged controls (> 50 years of age) participating in one of two prospective studies underwent [^18^F]FDG PET/MRI. All included controls were free of neurological and psychiatric disease, had no subjective memory complaints and had normal results on dementia screening tests including mini-mental state examination (MMSE) and Addenbrooke’s Cognitive Examination (ACE). Six participants were excluded due to abnormalities on MRI (pronounced atrophy *n* = 1, vascular pathology *n* = 3, meningioma *n* = 1), or subsequent diagnosis of mild cognitive impairment (MCI, *n* = 1), resulting in a final healthy aged control group of 38 participants. In addition, resting ASL scans from 12 young healthy male volunteers participating in a [^15^O]H_2_O validation study [7] were included to assess the effects of age on CBF and ASL image quality in healthy subjects. All healthy controls participated in studies approved by the local ethics committee (H-18035941, H-1–2014-126, and H-16023156) conducted in accordance with the Helsinki Declaration. All healthy participants provided written informed consent prior to the scan.

#### Patients

Between January 2019 and August 2019 consecutive memory clinic patients referred for [^18^F]FDG PET/MRI as a part of the diagnostic work-up for suspected cognitive impairment due to a neurodegenerative dementia disorder were invited to participate in the study. On the day of the scheduled scan the patients were asked to undergo a supplementary ASL sequence at the end of the examination. Patient inclusion was based on written and oral informed consent and ability to cooperate to the extended scan duration. Inclusion continued until scans from 100 unique patients had been completed. Ethical approval was waived by the local Ethics Committee and study of patients was conducted as a quality assurance study. Use of clinical data was approved by the Danish Patient Safety Authority (ref. 3–3013-3144/1).

### Scan protocol

PET/MRI imaging was done following 4 h of fasting. All scans were performed on a Siemens mMR hybrid PET/MR scanner (Siemens Healthineers AG, Erlangen, Germany) equipped with a 16-channel head and neck coil.

[^18^F]FDG PET scans were performed in accordance with international guidelines [13]. A 10 min emission scan was initiated approximate 40 min after the intravenous injection of 200 MBq of [^18^F]FDG. PET images were reconstructed into a 344 × 344 matrix (voxel-size 0.8.x0.8 × 2 mm^3^, zoom 2.5) using 3D OP-OSEM (4 iterations, 21 subset) and applying a 3 mm Gaussian filter. Attenuation correction was performed using µ-maps generated with a separately acquired low-dose CT scan as previously described [14] or a deep learning synthetic CT based on a Dixon MRI sequence [15].

The MRI protocol included as a minimum a high-resolution 3D T1 weighted and axial T2 FLAIR weighted sequences to exclude significant structural and ischemic vascular pathology. Scan parameters of the 3D T1 scan (MPRAGE) used for segmentations were repetition time (TR) 1900 ms, echo time (TE) 2.44 ms, flip angle 9 degrees, matrix 256 × 256, voxel size 1.0 × 1.0 × 1.0 mm^3^, GRAPPA acceleration factor 2. In patients also axial T2, and axial T2* weighted imaging was performed as a part of the standard clinical protocol.

The ASL sequences was a 2D PCASL adapted to a multi-PLD version by repeating the scan at different PLDs [7]. The labelling plane was placed across the neck 9 cm beneath the centre of the imaging slab and the labelling duration was 1800 msec. The acquisition parameters were: 21 slices, TE 12 msec, voxel size 4.0 × 4.0 × 6.0 mm^3^, field of view 256 × 256 mm^2^, TR was adjusted for each PLD in order to keep the scanning time as short as possible. The different PLDs were set at 200, 500, 800, 1100, 1400, 1700 and 2000 msec, acquired in ascending order. Six pairs of labelled-control images were acquired in each PLD. After each ASL scan, a single equilibrium magnetisation scan (M0) was acquired with the same parameters as the previously described ASL images except for a 10.000 msec TR. Total scanning time was seven minutes.

### Data analysis

ASL images were quantified using BASIL in FSL (FMRIB software library, www.fmrib.ox.ac.uk) with a full quantitative modelling of the Buxton kinetic model [16, 17]. Details of ASL analysis are provided in Suppl. Methods.

The MPRAGE was segmented using FreeSurfer (version 7.1.1, https://surfer.nmr.mgh.harvard.edu/). In four patients FreeSurfer segmentation was not able to complete, leaving 96 patients for the final analysis. Standard anatomical regions (from FreeSurfer Desikan-Killiany Atlas and lobe definition from [18]) corresponding to a large cerebral cortical regions (frontal, parietal, occipital and temporal cortex, and a combined cortex region), and smaller cortical regions (hippocampus, isthmus-cingulate, cuneus and precuneus) as well as cerebellar cortex were defined (see also Suppl. Methods and Suppl. Fig S1). Median region of interest (ROI) values were extracted for both ASL CBF (in ml/100 g/min) and [^18^F]FDG PET for each hemisphere separately and combined. ROI values were for [^18^F]FDG PET reported as standardised uptake value normalised to median cerebellar cortical value (SUVr). In addition, ROI values and parameters maps normalised to median global cortex value were calculated for both SUVr (SUV_glob_) and for CBF (CBF_glob_) to allow for semiquantitative comparison of regional correlation of metabolism and perfusion.

For visual reading a PDF page for each participant was created displaying a standardised presentation with five axial slices, three coronal slices and two parasagittal slices of ASL CBF (absolute CBF, scaled 0–100 ml/100 g/min) and FDG PET (SUVr scaled 0.4–1.6 a.u.) images with corresponding T1 MPRAGE slices shown for comparison (see Suppl. Fig. S2 for example). Images were shown in fixed position in Talairach orientation based on FreeSurfer registration of MPRAGE (affine transformation, 6 degrees of freedom).

Whole cortex intrasubject spatial coefficient of variation (sCOV) was calculated from mean and standard deviation values within the whole cerebral cortex as a quantitative measure of image quality [10].

### Visual assessment

**Fig. 1:**
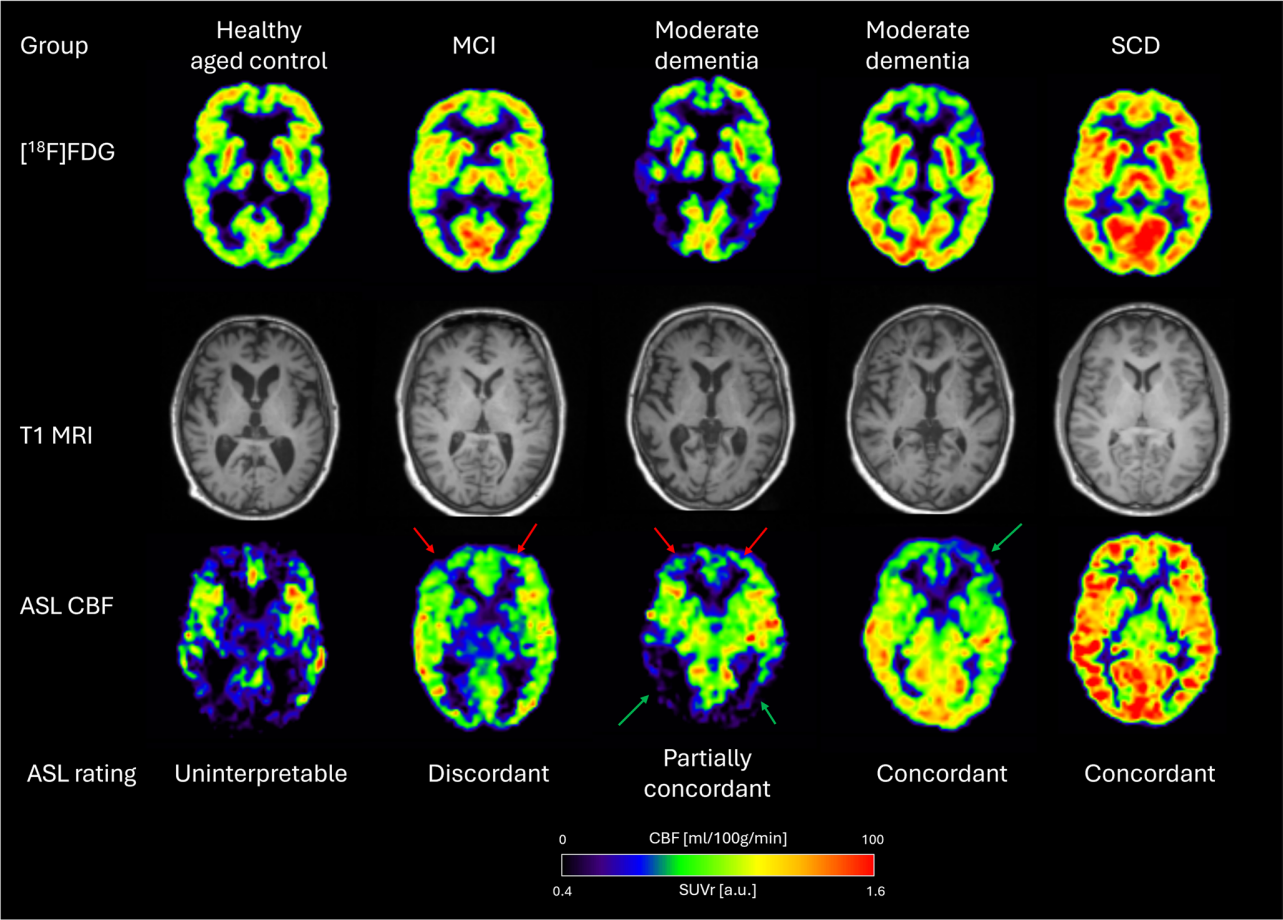
ASL rating of quality and concordance. Examples of T1 MRI and corresponding ASL CBF and [^18^F]FDG SUVr images. Arrows point to areas of regional discordance (red) and concordance (green)

**Fig. 2:**
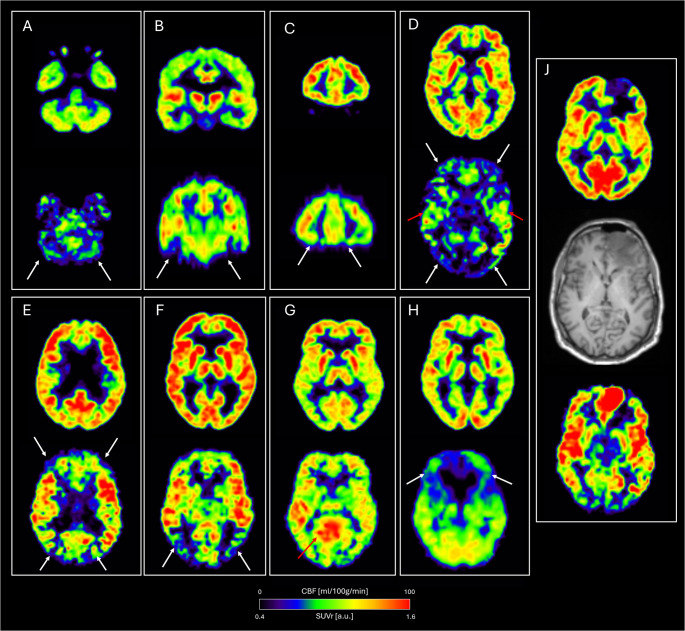
Examples of discordant ASL MRI CBF findings in healthy aged controls with normal [^18^F]FDG PET. For each example corresponding FDG PET (top) and ASL CBF (bottom) is shown using fixed scaling (as in Fig. 1). Arrows point to areas of apparent hypoperfusion (white) or hyperperfusion (red) with normal [^18^F]FDG uptake. **A-C** typical defects in basal structures due to susceptibility artefacts in EPI sequence (and not leading to rating as discordant), **D** global transit time artefacts causing widespread hypoperfusion and vascular high signal in insula, **E-F** symmetric hypoperfusion in watershed areas likely due to transit time effects, **G** hyperperfusion in posterior cingulate areas of unknown origin, **H** large area of pronounced hypoperfusion of unknown origin, **J** true high perfusion and low [^18^F]FDG uptake in large frontal meningioma found in healthy control subsequently excluded

For each set of images, two readers (OMH and OPC), independently and blinded to diagnosis assessed ASL image quality and level of concordance with [^18^F]FDG PET images from both healthy controls and patients. For healthy young controls with no [^18^F]FDG PET available, a normal [^18^F]FDG PET scan was assumed. Regional discordances are likely to represent *method* associated ASL artefacts due to susceptibility or transit time effects, but from these data it is not possible to reliably differentiate regional ASL artefacts from true *biology* associated hypo- or hyperperfusion. First, interpretability of ASL image quality was assessed and rated as *uninterpretable* in case of severe global artefacts prohibiting assessment of regional cortical patterns. Secondly, level of concordance with [^18^F]FDG PET was assessed as *discordant*, i.e. predominantly discordant abnormalities with severe or (also milder) large regional deviations likely to alter overall interpretation (e.g. from normal to abnormal), as *partially concordant*, i.e., mixed concordant/discordant abnormalities, or smaller regional deviations or recognisable artefacts with minor influence on interpretation, or as *concordant*, i.e. overall similar regional pattern and interpretation. Typical focal ASL artefacts in basal frontal and temporal structures and in cerebellum, which are known ASL limitations and present in vast majority of scans were not considered discordant. Scans rated as uninterpretable or discordant were considered *non-comparable* to [^18^F]FDG PET by providing no or potentially misleading information if used as a surrogate marker of hypometabolism in a MRI only setting. Examples of ratings are provided in Fig. 1 and examples of discordant findings in Fig. 2. In case of disagreement between readers, a final score was determined by consensus. For the purpose of statistical analysis, image quality and concordance rating were combined into a single ordinal ASL applicability scale reflecting potential clinical information compared to [^18^F]FDG PET (1 = uninterpretable, 2 = discordant, 3 = partially concordant and 4 = concordant).

### Normal database and Z-scores statistics

From the healthy aged controls, a normal database was created to produce ASL and FDG Z-score statistics. Scans from a participant with uninterpretable ASL (score = 1) and from a participant with a large frontal artefact (Fig. 2, panel H) were not included in the normal database. Voxel-wise Z-scores (the deviation from the database mean value divided by the database standard deviation) in MNI space were calculated in order to generate single subjects Z-score maps for illustration of regional abnormalities of CBF_glob_ and SUV_glob_. Details of image registration for Z-score analysis are provided in Suppl. Methods.

**Fig. 3:**
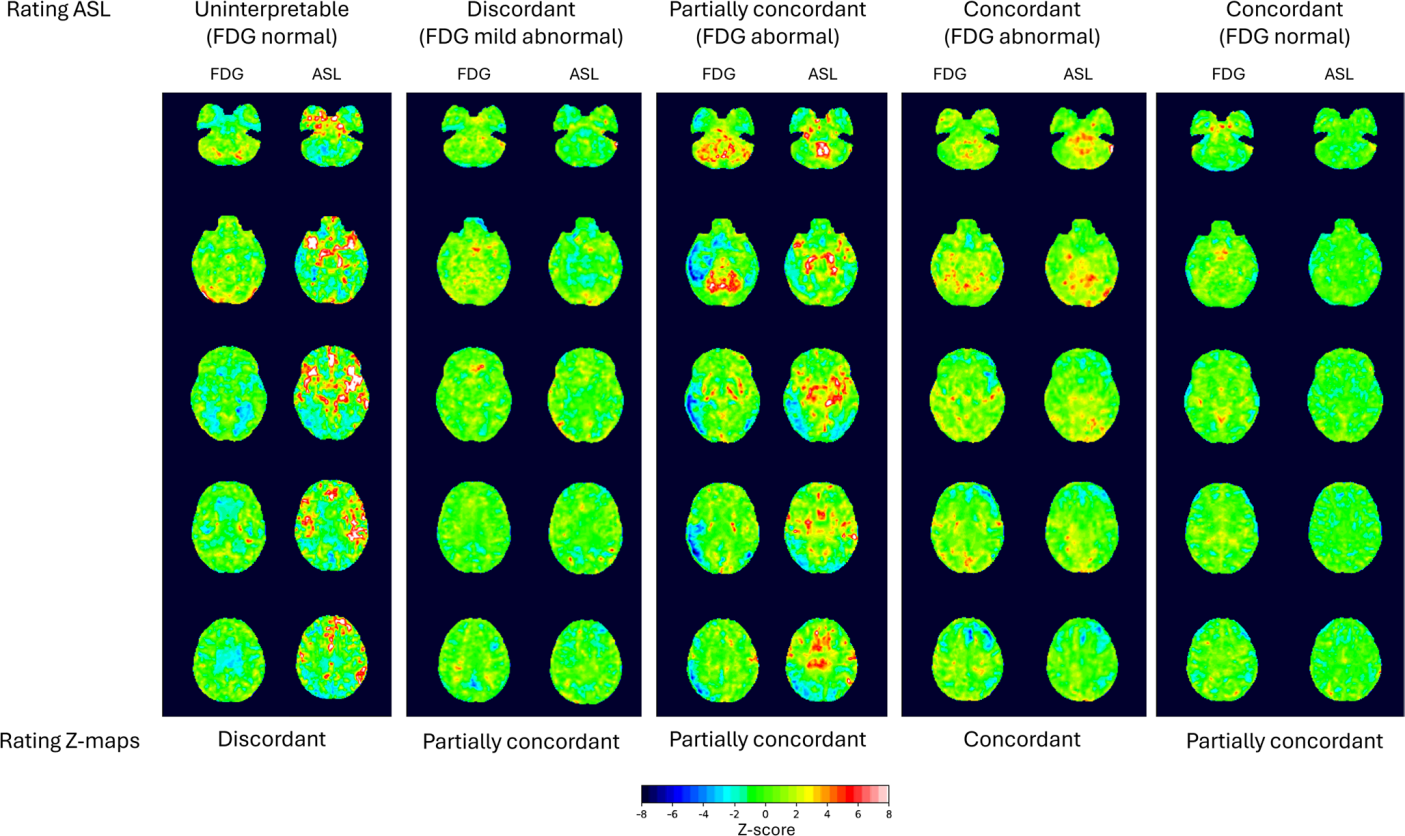
Examples of Z-score maps. Comparison of [^18^F]FDG PET and ASL Z-score based parameter maps. Five axial slices are shown for same subjects as in Fig. 1. Note areas of positive Z-scores (yellow/red) as a result of normalisation to whole cortex mean. Areas of negative Z-scores (blue) tends to be larger in [^18^F]FDG PET maps than ASL indicating higher sensitivity

Examples of Z-score maps are shown in Fig. 3. Z-score maps for CBF_glob_ and SUV_glob_ from all patients and aged controls were first compared and rated as providing *discordant*, *partially concordant* or *concordant* information on abnormalities (negative Z-values). Subsequently readers should assess if ASL Z-score maps improved concordance of ASL with FDG PET, i.e. as worse (e.g. supressing true abnormalities), equal, or better (supressing false abnormalities). Finally, for each Z-score map volume of voxels with Z < −2.0 was calculated as a measure of extent of hypometabolism or hypoperfusion.

### Clinical information

The patient records were reviewed in the patient group by two experienced memory clinic specialists (SGH and KSF). For each patient MMSE, ACE, level of cognitive impairment (*subjective cognitive decline* (*SCD*), *MCI*, *mild dementia*, *moderate dementia* or *severe dementia* according to ICD-10) and final clinical diagnosis were noted. Also, presence of small vessel (lacunar infarcts or a Fazekas score ≥ 2) or large vessel disease (non-lacunar infarcts) on MRI were noted.

### Statistics

All continuous parameters are reported as median [range] unless stated otherwise. Group differences were investigated by one-way ANOVA for continuous parameters. Distribution of categorical variables were assessed by chi^2^ test of n x n contingency tables. Factors associated with ASL applicability score was investigated by ordinal logistic regression models. Optimal ASL CBF sCOV cut-off for separation of uninterpretable and interpretable scans was determined by receiver operation characteristics (ROC) analysis and maximisation of Youden’s index.

Associations of ASL CBF with [^18^F]FDG SUVr were initially investigated by scatterplots and calculation of crude correlation (Pearson’s R^2^) for each ROI including values from both hemispheres from each subject. To account for the multilevel data structure (two hemispheres per scan), 2-level mixed linear models were investigated for each ROI with ASL CBF as outcome, [^18^F]FDG SUVr as fixed effect, and subject as random effect. Pooling data from healthy aged controls and patients both models with absolute CBF vs. SUVr, and CBF_glob_ vs. SUV_glob_ were analysed with interpretable and uninterpretable scans analysed separately.

## Results

Demographics and characteristics of healthy controls and patients (and patient groups) are summarised in Table 1.

**Table 1:** Summary statistics all participants

	HC-young *n* = 12	HC-aged *n* = 38	SCD *n* = 13	MCI *n* = 27	Mild *n* = 40	Moderate *n* = 16	*p*-val
Age, year	23 [21–28]	69.5 [54–81]	55 [39–69]	70 [46–86]	75 [50–88}	79 [64–85]	< 0.001 ^a^
MMSE	-	29.5 [25–30]	30 [19–30]	28 [22–30]	24 [14–30]	18.5 [13–27]	< 0.001 ^a^
AD diagnosis	-	-	-	7 (25.9%)	21 (52.5%)	8 (50.0%)	0.091^b^
[^18^F]FDG SUVr global	-	1.13 [1.01–1.28]	1.17 [0.97–1.23]	1.06 [0.89–1.20]	0.99 [0.85–1.16]	0.94 [0.85–1.17]	< 0.001 ^a^
Fazekas 0/1/2/3, n	12/0/0/0	17/20/1/0	11/1/1/0	10/8/4/5	9/18/5/8	1/9/2/4	< 0.001 ^b^
ASL rating and quality							
Uninterpretable, n (%)	0 (0%)	2 (5.3%)	0 (0%)	1 (3.7%)	10 (25%)	7 (43.8%)	< 0.001 ^b, c^
Discordant	1 (8.3%)	12 (31.6%)	2 (15.4%)	15 (55.6%)	10 (25%)	6 (37.5%)	
Partially concordant	5 (41.7%)	19 (50%)	6 (46.2%)	10 (37.0%)	14 (35%)	2 (12.5%)	
Concordant	6 (50%)	5 (13.2%)	5 (38.5%)	1 (3.7%)	6 (15%)	1 (6.3%)	
Whole cortex sCOV	0.37 [0.31—0.43]	0.39 [0.29–0.80]	0.37 [0.28–0.50]	0.46 [0.35–1.13]	0.48 [0.29–0.94]	0.62 [0.39–1.07]	< 0.001 ^a^
sCOV > 0.59, n (%)	0 (0%)	2 (5.3%)	0 (0%)	4 (14.8%)	16 (40%)	10 (62.5%)	< 0.001 ^b^
Whole cortex ASL CBF (ml/100 g/min)							
All scans	79.0 [64.8–92.3]	55.7 [24.4–109.4.4.4]	66.9 [44.8–102.3.8.3]	54.1 [20.3–73.5]	44.6 [22.0–81.4.0.4]	35.4 [10.3–60.5]	< 0.001 ^a^
Interpretable (score > 1)	79.0 [69.0–92.3.0.3]	55.9 [39.8–109.4.8.4]	66.9 [48.8–102.3.8.3]	54.9 [38.8–73.5]	51.4 [25.1–81.4]	41.4 [31.2–60.5]	< 0.001 ^a^
Uninterpretable (score = 1)	-	25.5 [24.4–26.7] †	-	20.3 [20.3–20.3]	31.5 [22.0–38.2.0.2] ‡	24.9[10.3–36.2] †	0.157 ^a^

† *p* < 0.05 and ‡*p* < 0.001 for uninterpretable vs. interpretable ASL quality scans, ^a^ one-way ANOVA, ^b^ chi^2^ test, ^c^ across all scores and groups, HC healthy controls, SCD subjective cognitive decline, MCI mild cognitive impairment, MMSE mini-mental state examination, AD Alzheimer’s disease, sCOV spatial coefficient of variation, CBF cerebral blood flow, SUVr standardised uptake value normalised to cerebellum

### Image quality and concordance

Visual image quality was highly dependent on subject group, with fraction of uninterpretable ASL scans increasing from none in young healthy volunteers and SCD, to ≤ 5% in healthy aged controls and MCI patients, while scans of 25% of patients with mild dementia and of 43.8% of patients with moderate dementia were considered uninterpretable. A total of 51 (53.1%) of ASL scans from patients (and 81.2% of patients with moderate dementia) were either uninterpretable or discordant when compared to [^18^F]FDG PET and, thus, considered non-comparable. Concordance was observed only in 13.5% of patients, and more frequently in patients with subjective cognitive impairment (38.5%) than in those with cognitive impairment (9.6%).

**Fig. 4:**
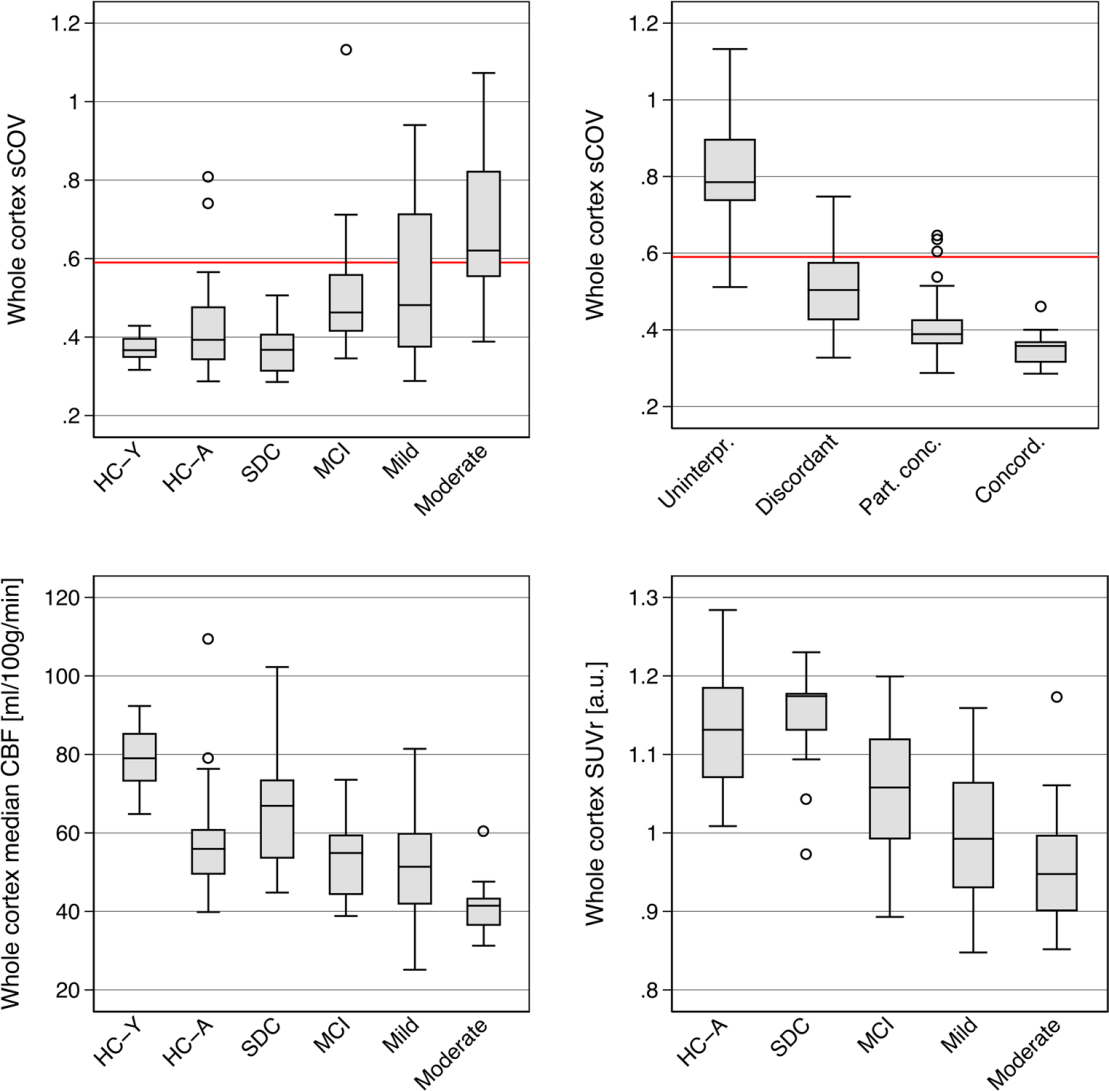
Boxplot of whole cortex ASL CBF sCOV, ASL CBF and [^18^F]FDG SUVr values across subject groups. For sCOV also distribution across ASL quality rating is shown. Red horizontal line shows optimal sCOV cut-off (0.59) for separation of uninterpretable scans. For ASL CBF only values from interpretable scan are included

Whole cortex ASL CBF sCOV varied across groups of healthy controls and patient categories and increased with increasing age and disease severity (Table 1; Fig. 4, and Suppl. Fig S3 for mean group images). Across all ASL scans sCOV was inversely and strongly associated with ASL applicability score (*p* < 0.001 by ordinal logistic regression). Whole cortex sCOV value was highly accurate for identification of uninterpretable scans (ROC AUC 0.98) and a value of 0.59 separated uninterpretable from interpretable ASL scans with a sensitivity of 95% and a specificity of 90%.

In patients, increasing whole cortex ASL CBF sCOV and decreasing ASL applicability score was predicted by increasing age (*p* < 0.001 for both), presence of small vessel disease (*p* = 0.001 and *p* = 0.016, respectively), increasing Fazekas score (*p* < 0.001 and *p* = 0.004, respectively), increasing disease severity (*p* < 0.001 for both) and decreasing [^18^F]FDG SUVr (*p* = 0.004 and 0.023, respectively), but not by large vessel disease or MMSE. Uninterpretable ASL CBF was predicted by increasing patient age (*p* = 0.006) and disease severity (*p* = 0.001), but not by small or large vessel disease, Fazekas score, MMSE or whole cortex [^18^F]FDG SUVr. In a model with both age and disease severity, only disease severity remained statistically significant (*p* = 0.011).

### Association of ASL CBF with [^18^F]FDG SUVr

**Fig. 5:**
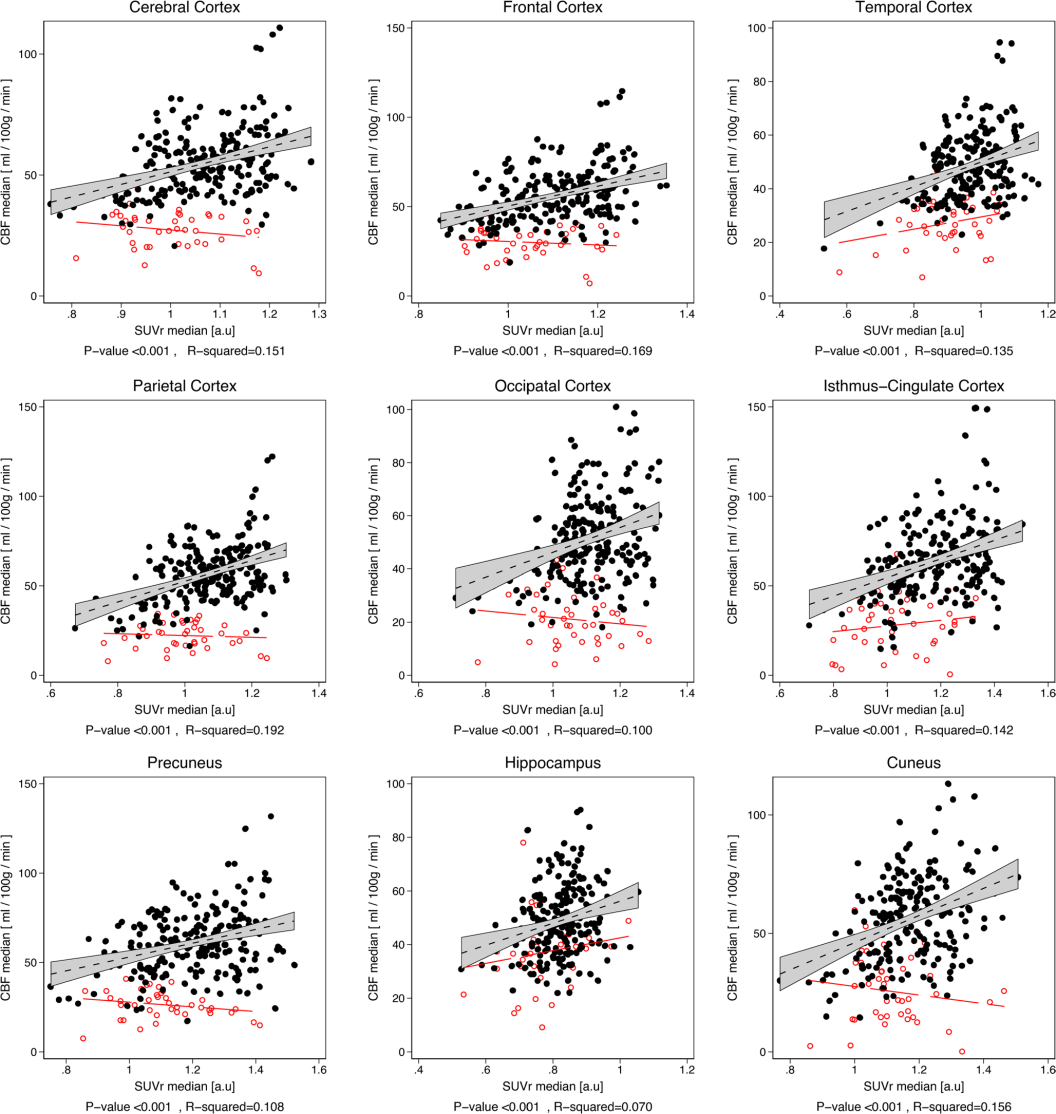
Association of absolute ASL CBF with [^18^F]FDG SUVr. Scatterplots of hemisphere values in participants (36 healthy aged controls and 78 patients) with interpretable ASL scans (black filled circles, *n* = 228) and uninterpretable ASL scans (red hollow circles, *n* = 40) with corresponding regression lines are shown for each region. R^2^ and p-value for interpretable quality scans are shown. For uninterpretable quality scans no statistically significant associations of absolute CBF with SUVr could be demonstrated (*p* > 0.05 for all)

Crude regional associations of ROI median absolute ASL CBF and [^18^F]FDG SUVr are shown in scatter plots in Fig. 5 and in Suppl. Table S1. In interpretable scans all associations between ASL CBF and [^18^F]FDG SUVr were highly significant (*p* < 0.001) although, in general, relatively weak with R^2^ < 0.2 for all. Strength of association improved for large cortical regions when comparing global normalised values (Suppl.Table S1 and Suppl. Fig S4). In general associations tended to be poorer in occipital cortex and variable for small cortical regions. Similar observations were made when applying mixed linear models and calculating marginal R^2^ (Table 2).

**Table 2:** Linear mixed model of regional association of ASL CBF and [^18^F]FDG SUVr (only interpretable ASL, *n* = 228 hemispheres from 114 subjects^§^)

			CBF vs. SUVr					CBF_glob_ vs. SUV_glob_	
			Beta †	pR^2^				Beta	pR^2^
Hemisphere cortex			50.9***	0.151				1.173	0.282
Large regions									
Frontal			59.8***	0.168				1.410****	0.289
Temporal			34.7***	0.126				0.902***	0.219
Parietal			56.0***	0.192				1.277***	0.212
Occipital			47.5***	0.100				0.836***	0.092
Small regions									
Cingulate			51.0***	0.142				1.103***	0.137
Precuneus			36.4***	0.107				0.758***	0.088
Hippocampus			38.3***	0.070				1.025***	0.185
Cuneus			54.0***	0.156				0.994***	0.110

*** *p* < 0.001, **p < 0.01, *p < 0.05, † ml/100 g/min per a.u. increase in SUVr, pR^2^ pseudo R2 calculated from variance component models

§ 36 healthy aged controls and 78 patients

### Z-score maps

Of 132 rated Z-score maps from aged volunteers and patients, 45 (34%) were rated as discordant, 63 (47%) as partially concordant, and 24 (18%) as concordant (Fig. 3; Table 3.). Adding Z-score maps improved concordance of ASL MRI with [^18^F]FDG PET in 46 (35%) of the scans. Concordance of Z-score maps and impact on agreement were closely associated with ASL score (*p* < 0.001 by Chi2 test). Thus, concordance of Z-maps was observed in 2/45 (4%) of discordant scans, in 14/50 (28%) of partially concordant scans, and in 8/18 (44%) of concordant scans. ASL Z-score were rated “better” in 30/50 (60%) in partially concordant scans, but only in 4/18 (11%) concordant scans, 14/45 (31%) of discordant scans, and none of the uninterpretable scans.

**Table 3.** Rating Z-score maps (*n* = 132) - healthy aged controls and patients

	Concordance Z-ASL vs. Z-FDG			Impact Z-ASL			
ASL rating	Discordant	Partially concordant	Concordant	Worse	Equal	Better	Total
Uninterpretable	18	1	0	0	19	0	19
Discordant	23	20	2	1	30	14	46
Partially concordant	3	33	14	2	18	30	50
Concordant	1	9	8	5	13	2	18
Total	45	63	24	8	80	46	132

Volume of voxels with Z-score <−2 was higher for [^18^F]FDG PET compared to ASL MRI in both concordant (36.5 ml vs. 22.0 ml, *p* < 0.001) and partially concordant scans (40.1 vs. 34.9 ml, *p* < 0.001), while the opposite was observed for discordant scans (47.1 ml vs. 88.5 ml, *p* < 0.001).

## Discussion

The present study is the first to investigate the potential of multi-PLD PCASL in unselected prospectively referred memory clinic patients with [^18^F]FDG PET as reference and also one of the largest head-to-head comparisons of ASL and [^18^F]FDG overall. The present study differs from previous studies by attempting to critically appraise the information provided by ASL by comparing regional patterns of perfusion and metabolism at the single subject level in a real life mixed clinical population. The main findings are that image quality of ASL CBF maps was overall poor with a high frequency of scans with either uninterpretable image quality or discordance prohibiting or potentially misleading clinical reading compared to [^18^F]FDG PET in 37% of healthy aged controls and 59% of patients with cognitive impairment. Based on these findings, multi-PLD PCASL as applied in the present study does not appear to overcome limitations of single-PLD approaches, and does not present as a viable alternative to [^18^F]FDG PET in memory clinic patients.

Several prior studies have compared [^18^F]FDG and single-PLD PCASL in memory clinic patients. The studies have in general relied on group comparisons by voxel-based analysis or by ROI based analyses in predefined disease specific populations (e.g. AD or FTD) vs. healthy controls, and assessing diagnostic performance by ROC analysis of ROI based metrics, but not visual reading. Two large studies of similar population size have been published. An analysis of ADNI data [19] from 87 patients with AD/AD-MCI and 34 healthy controls concluded that ASL (sequence not specified) had similar diagnostic performance as [^18^F]FDG PET when assessing regional patterns by partial least squares regression analysis. It should be noted that ADNI applies strict inclusion criteria [20] and the study population is therefore not comparable to a real-life clinical population with significant comorbidities and less clear presentations. A more recent single centre study [21] included 47 healthy controls, 45 aMCI and 45 AD patients undergoing PET/MRI with single-PLD 3D PCASL. Using ROI derived metrics ASL showed consistently lower accuracy (ROC AUC) for separating diagnostic groups. Neither of the two studies included visual assessment or reported frequency of poor image quality. Due to the overall poor image quality and the mixed population, we did not attempt to assess diagnostic value, but relied on a simplified visual comparison of the information provided by ASL and [^18^F]FDG PET. Visual reading has only been performed in smaller studies, of which the largest reported lower sensitivity and observer agreement of ASL compared to [^18^F]FDG PET in 27 mixed memory clinic patients when analysed by visual reading, but similar performance of ROI based metrics [22]. These prior observations together with our findings highlight that group and ROI based analysis in highly selected patient groups may inflate the ability of ASL to reliably detect neurodegenerative patterns in a clinically relevant population, and that visual assessment is imperative when evaluating clinical usefulness of ASL.

For reading of [^18^F]FDG PET comparison with normal database and generation of Z-score maps has been shown to improve diagnostic accuracy [23] and reader agreement, and is generally accepted as an valuable part of clinical reading of brain [^18^F]FDG PET, in particular for assessment of early disease stages with more subtle changes in metabolism. Similar advantages could apply to ASL imaging and could for comparison of modalities further take into account modality specific regional variations. A previous study reported higher specificity, but lower sensitivity of ASL Z-score statistics when comparing [^18^F]FDG PET for separation of patients from healthy controls [24]. In our study application of Z-score maps tended to improve concordance of modalities, mainly in partially concordant scans, but with lower extent of abnormalities in concordant Z-score maps. These observations suggest that Z-score may only be of value in good quality scans and less sensitive when compared to [^18^F]FDG PET. This lower performance of ASL Z-score maps most likely reflect the higher variability in the ASL normal database. Also, the need for reliable reference region is of key importance as has been shown for [^18^F]FDG PET [25]. Due to highly variable ASL quality in infratentorial structures, the cerebellum typically used for [^18^F]FDG PET proved unreliable in exploratory analyses, and whole cortex mean was applied resulting in *false* apparent hyperperfused foci. Thus, calculation of Z-score maps may facilitate clinical reading of single subject ASL, but in our implementation did not overcome challenges of uninterpretable ASL scans or lack of reliable reference regions.

In addition to visual rating of agreement with FDG, spatial sCOV was calculated as a quantitative measure of image quality [10]. A recent large study applying single-PLD 3D pulsed ASL (PASL) in a mixed population demonstrated similar high frequency of artefacts related to prolonged ATT with severe artefacts (equivalent to “uninterpretable” in the present study) in 28% of participants and moderate artefacts in an additional 37% unrelated to disease severity or vascular disease burden, and similar to the present study found as strong association of ASL CBF sCOV with visual image quality supporting sCOV as proxy of ATT [26].

Although glucose metabolism and perfusion are tightly coupled in the brain, the two represents different physiological properties. As [^18^F]FDG is continuously accumulated by active transport over more than 1 h with imaging after 40 min and is less influenced by CBF. Glucose metabolism may thus be relatively preserved in areas with reduced CBF and we cannot rule out true hypoperfusion as a cause of discordance. Regional perfusion-metabolism mismatch has indeed been reported in e.g. epilepsy [27] and chronic steno-occlusive disease (using single-PLD ASL) [28] or in certain anatomical regions [29], but to our knowledge there is very limited evidence from studies applying reference techniques to support such an effects in patients milder small vessel disease as in the present sample. AD related regional uncoupling of perfusion and glucose metabolism has been suggested to explain differences between ASL CBF and [^18^F]FDG PET imaging [19], but findings from studies applying gold standard PET techniques for CBF quantification do not support hypoperfusion exceeding hypometabolism as a typical AD feature [30, 31]. Accordingly we find it unlikely that this would account for the majority of discordant findings in the absence of significant cerebrovascular disease, and discordance may in our opinion primarily be attributed to limitations and errors of ASL.

From a technical point of view, it is somewhat disappointing that a validated multi-PLD approach performed so poorly when applied to a clinically relevant population. Even in healthy aged controls free of cerebrovascular or neurological disease, discordant abnormalities were frequent. Most likely, the range of PLDs did not fully cover the range of ATT values in aged participants and those with dementia. In order to use the validated sequence settings, we used the same imaging parameters as applied in the validation study including seven PLDs from 200 to 2000 msec (labelling duration of 1800 msec). The choice of parameters agreed with PCASL recommendations in elderly subjects at the time of the study [5], while in the recent multi-PLD recommendations a wider PLD range is suggested in older and clinical subjects (five PLDs from 200 to 2800 msec and a labelling duration of 2050 msec). Analysis of multi-PLD data can provide ATT estimates, but ATT values provided are unreliable as only a part of the signal curve is measured, and model fitting may be grossly erroneous if capturing mainly the ascending or descending part of the curve. Consequently, from our data we cannot assess the impact of ATT on level of concordance. Including later PLDs could have improved detection of very prolonged ATTs, but at the expense of lower SNR, in particular for later PLDs, which may add to errors of kinetic modelling. SNR at long PLDs may be improved by prolonging labelling duration as in the long-label long-delay approach [32]. 3D acquisition greatly improves SNR of PCASL and would potentially also increase the value of long PLDs. However, patient movement in elderly persons and patients with dementia poses an additional challenge in ASL when subtracting image pairs, and may in fact be more severe using 3D than faster 2D imaging. Irrespective of the source of errors, the findings demonstrate that advanced MRI techniques may perform better in a typical research population of young healthy individuals, than in a clinical setting including aged patients with co-morbidities and more prone to movement.

In the literature, there is very limited experience with multi-PLD approaches in memory clinic patients. A study aiming to optimise automated processing of multi-PLD (100–2100 msec) 2D PCASL data reported an improvement in SNR when discarding poor subtraction pairs, e.g. due to head movement, but still a close, negative correlation of SNR from 1.5T data and similarity with regional CBF assessed by [^99m^Tc]HMPAO or [^99m^Tc]ECD SPECT was reported in 60 patient with MCI or dementia [33]. As opposed to standard multi-PLD, time-encoded (Hadamard) PCASL allows for sampling of the entire signal curve including also bolus arrival and thus potentially more accurate quantification of both CBF and ATT. Studies comparing time-encoded PCASL with single-PLD 3D PCASL have reported higher sensitivity of time encoded ASL for detecting CBF changes in 59 subjects across early AD spectrum [34], but similar performance for detection of regional hypoperfusion in nine FTD patients using [^15^O]H_2_O PET as reference [35]. An alternative approach using PASL and applying a look-locker multi TI sampling strategy combined with crusher gradients permits model-free analysis and showed in a small study of 13 AD patients regional hypoperfusion in AD relevant regions when compared to healthy controls [36]. Overall, these studies lend some support to the use of multi-PLD approaches, but studies demonstrating superior clinical performance to single-PLD are lacking.

Due to the high frequency of uninterpretable scans observed in 19% of patients overall (19%) and 30% of patients with mild-to moderate dementia, radiologists may be reluctant to prolong the standard dementia MRI protocol to obtain ASL. Even in interpretable ASL scans, the frequency of discordant findings was high, with some level of discordance observed in 83% of patients (and 75% in healthy aged controls). Thus, it would be difficult to interpret abnormalities, as areas of low perfusion may be very likely to demonstrate preserved metabolism on [^18^F]FDG PET. Consequently, only a normal scan may be considered of clinical value by predicting normal [^18^F]FDG PET (as in the SCD group), whereas abnormalities other than well-known recognisable artefacts would prompt the need for further investigation, e.g. [^18^F]FDG PET.

In terms of study design, the advantages of the present study over prior studies include the use of a validated multi-PLD ASL sequence, simultaneous acquisition of PET and ASL using PET/MRI, inclusion of a large clinically relevant mixed memory clinic population and visual reading at the single subject level. However, a number of limitations should be addresses. First of all, at the time of the study we did not have access to a state-of-art 3D sequence but relied on 2D sequence adapted for multi-PLD acquisition leading to lower SNR and limitations in terms of PLD range. Secondly, due to time constraints in a clinical setup, we did not include alternate ASL schemes for comparison, and we cannot make inferences about the relative performance of the two strategies. Finally, absolute CBF measurements by ASL were compared with relative glucose metabolism and not a CBF reference technique such as [^15^O]H_2_O PET which, however, was considered neither practical nor relevant for clinical practice.

## Conclusions

The multi-PLD PCASL scheme investigated showed overall poor image quality and did not provide diagnostic information comparable to [^18^F]FDG PET in more than half of patients when applied to a mixed memory clinic population. Although highly significant association of semiquantitative measures were observed, interpretation of apparent perfusion abnormalities is unreliable and cannot be used for patient diagnosis in a MRI only setting. Comparative studies of 3D PCASL with longer PLD are warranted.

## Supplementary Information

Below is the link to the electronic supplementary material.

Supplementary Material 1 (DOCX 2.45 MB)

## Data Availability

Data used and/or analysed during the current study are available from the corresponding author on reasonable request.
