# Supplementary material for: Simultaneous multiple post-labelling delay ASL MRI and [18F]FDG PET in a mixed memory clinic population and healthy controls
Source: Eur J Nucl Med Mol Imaging. 2026 Jan 12;53(5):3408–21. doi: 10.1007/s00259-025-07736-8 (PMC13013397; doi:10.1007/s00259-025-07736-8)
Supplement: Supplementary file 1 — Supplementary Material 1 (DOCX 2.45 MB) [file 259_2025_7736_MOESM1_ESM.docx]

**Supplementary Information**

Simultaneous multiple post-labelling delay ASL MRI and [^18^F]FDG PET in a mixed memory clinic population and healthy controls

Authors

Otto M. Henriksen (0009-0004-0472-015X) ^1^, Oriol P. Calvo (0000-0002-3510-9204) ^1,2,5^, Frederik J. Bruun (0000-0002-9054-054X) ^1^, Marie Bruun (0000-0002-1066-1035) ^3,5^, Steen G. Hasselbalch (0000-0003-4750-4911) ^3,5^, Kristian S. Frederiksen (0000-0001-5124-4417) ^3,5^, Adam E. Hansen (0000-0002-6457-1537) ^4,5^, Ian Law (0000-0001-9644-7496) ^1,5^, Ulrich Lindberg (0000-0002-0004-6354) ^1^

^1^ Dept. of Clinical Physiology and Nuclear Medicine, Copenhagen University Hospital - Rigshospitalet, Copenhagen, Denmark

^2^ Dept. of Nuclear Medicine, Zealand University Hospital, Køge, Denmark

^3^ Dept. of Neurology, Danish Dementia Research Centre, Copenhagen University Hospital - Rigshospitalet, Copenhagen, Denmark

^4^ Dept. of Radiology, Copenhagen University Hospital - Rigshospitalet, Copenhagen, Denmark

^5^ Dept. of Clinical Medicine, Faculty of Health and Medical Science, University of Copenhagen, Copenhagen, Denmark

Corresponding author: Otto M. Henriksen, e-mail: otto.moelby.henriksen.01@regionh.dk

*Supplementary Methods*

ASL processing

Data processing of the arterial spin labelling data was carried out as in Puig et al.[1] including some additional steps for data cleaning and without correcting T1 for individual haematocrit as these were not acquired. Within each post labelling delay, image pairs of bad quality were automatically removed using *fsl_motion_outliers* (part of FSL). M0 calibration was fitted on the control images using the saturation recovery equation also giving the R1 map. Voxel-wise analysis was carried out using *oxford_asl* (part of FSL) with standard parameters for pseudo continuous data and with voxel-wise quantification using the abovementioned M0 map.

Region of interest analysis

Region of interests were delineated on the high resolution T1-weighted anatomical MR using using the combined volumetric and surface based (CVS) registration algorithm (Postelnicu-Zollei-Fischl, TMI09) within *mri_cvs_register* (part of FreeSurfer).

Z-score map

PET data were registered to the high resolution T1-weighted image using a rigid transformation. Arterial spin labelling data was registered to the high resolution T1-weighted image using the fitted R1 map as input to the boundary-based registration algorithm, *bbregister* (part of FreeSurfer) [2]. The individual transformations where combined with the T1w-to-standard space non-linear transformation and applied to yield resulting SUV_glob_ and CBF_glob_ maps in standard space.

References :

1.Puig O, Henriksen OM, Vestergaard MB, Hansen AE, Andersen FL, Ladefoged CN, et al. Comparison of simultaneous arterial spin labeling MRI and (15)O-H2O PET measurements of regional cerebral blood flow in rest and altered perfusion states. J Cereb Blood Flow Metab. 2019:271678X19874643. doi:10.1177/0271678X19874643 [doi].

2. Greve DN, Fischl B. Accurate and robust brain image alignment using boundary-based registration. Neuroimage. 2009;48:63-72. doi:10.1016/j.neuroimage.2009.06.060.

**Supplementary figures and tables**

**Suppl. Table S1.** Crude regional associations of ASL CBF with [^18^F]FDG SUVr (only interpretable ASL, n=228 hemispheres from 114 scans^§^)

|  | CBF vs SUVr | |  |  | CBF_glob_ vs SUV_glob_ | |
| --- | --- | --- | --- | --- | --- | --- |
|  | Beta | R2 |  |  | Beta | R2 |
| Hemisphere cortex | 51.8*** | 0.151 |  |  | - | - |
| Large regions |  |  |  |  |  |  |
| Frontal | 55.5*** | 0.169 |  |  | 1.351*** | 0.286 |
| Temporal | 46.0*** | 0.135 |  |  | 1.026*** | 0.222 |
| Parietal | 57.8*** | 0.192 |  |  | 1.371*** | 0.213 |
| Occipital | 46.6*** | 0.100 |  |  | 0.856*** | 0.092 |
| Small regions |  |  |  |  |  |  |
| Isthmus-Cingulate | 51.7*** | 0.142 |  |  | 1.088*** | 0.137 |
| Precuneus | 38.6*** | 0.108 |  |  | 0.890*** | 0.090 |
| Hippocampus | 41.1*** | 0.070 |  |  | 1.213*** | 0.190 |
| Cuneus | 57.3*** | 0.156 |  |  | 1.015*** | 0.110 |

*** p< 0.001, **p<0.01, *p<0.05, § from 36 healthy aged controls and 78 patients.


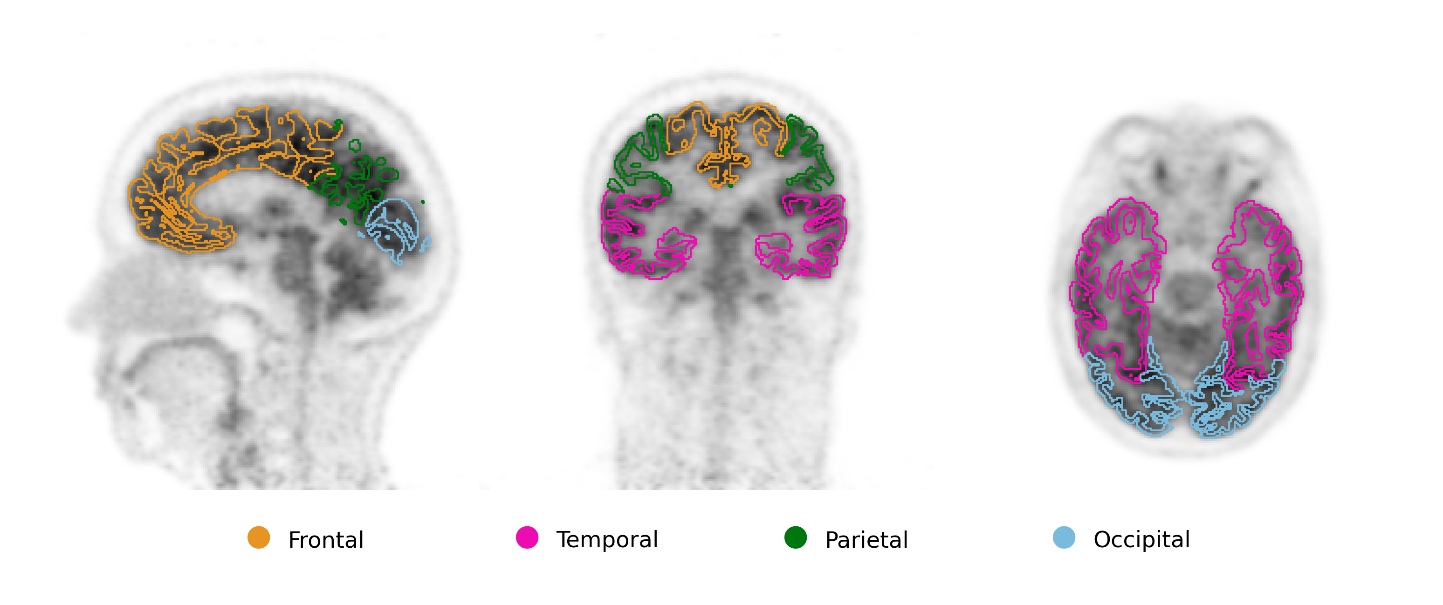


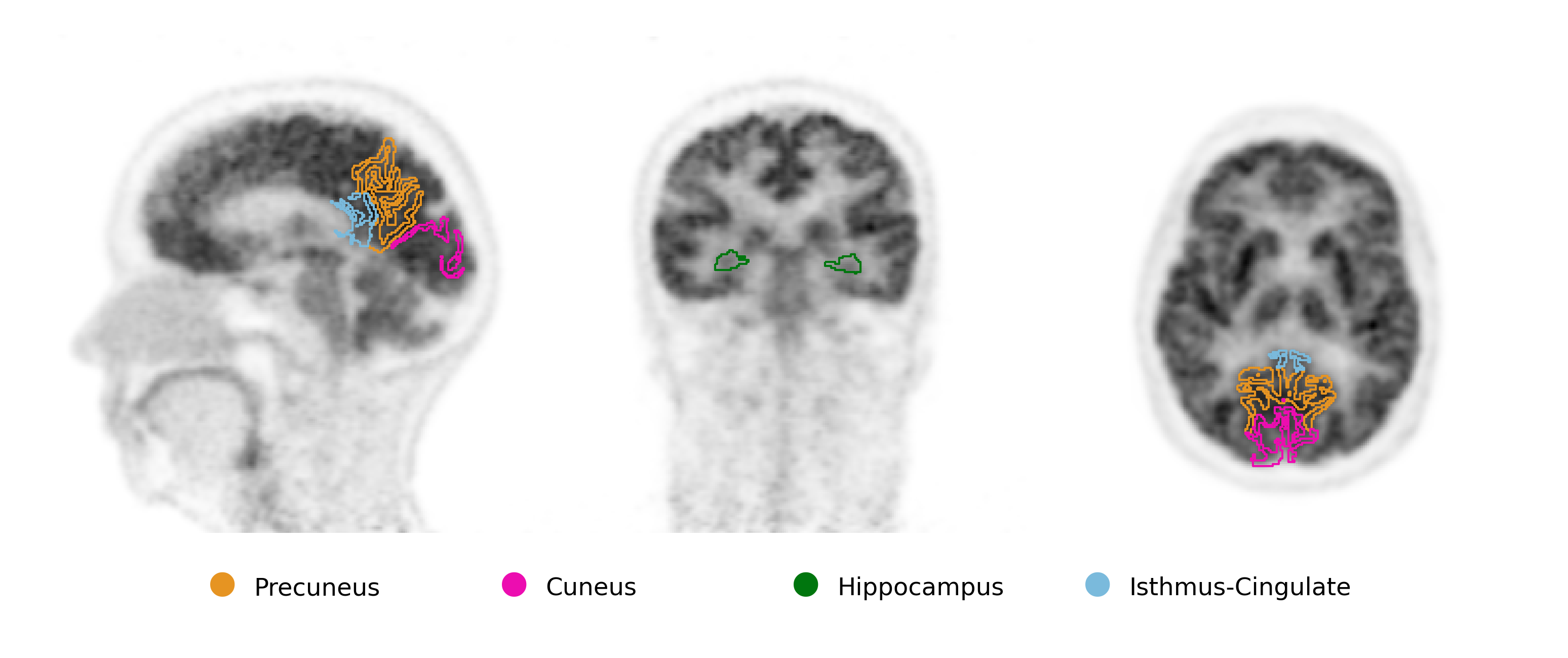


**Suppl. Fig. S1.** Anatomical regions of interest superimposed on [^18^F]FDG PET image from healthy aged control.


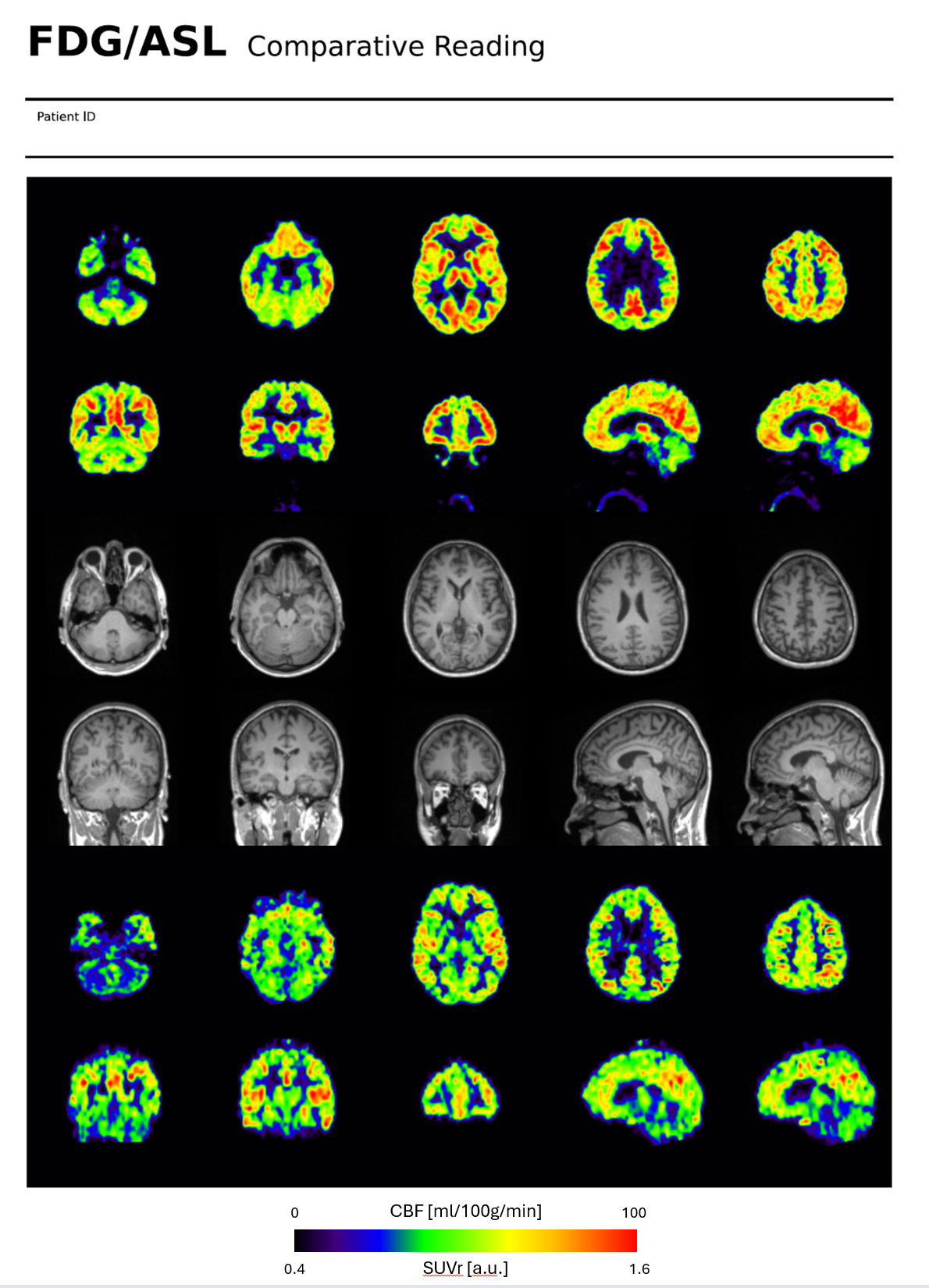


**Suppl. Fig. S2.** Example PDF report. Corresponding axial, coronal and sagittal views of (top), 3D T1 MRI (mid), and ASL CBF (bottom) are shown using fixed colour scales.

**
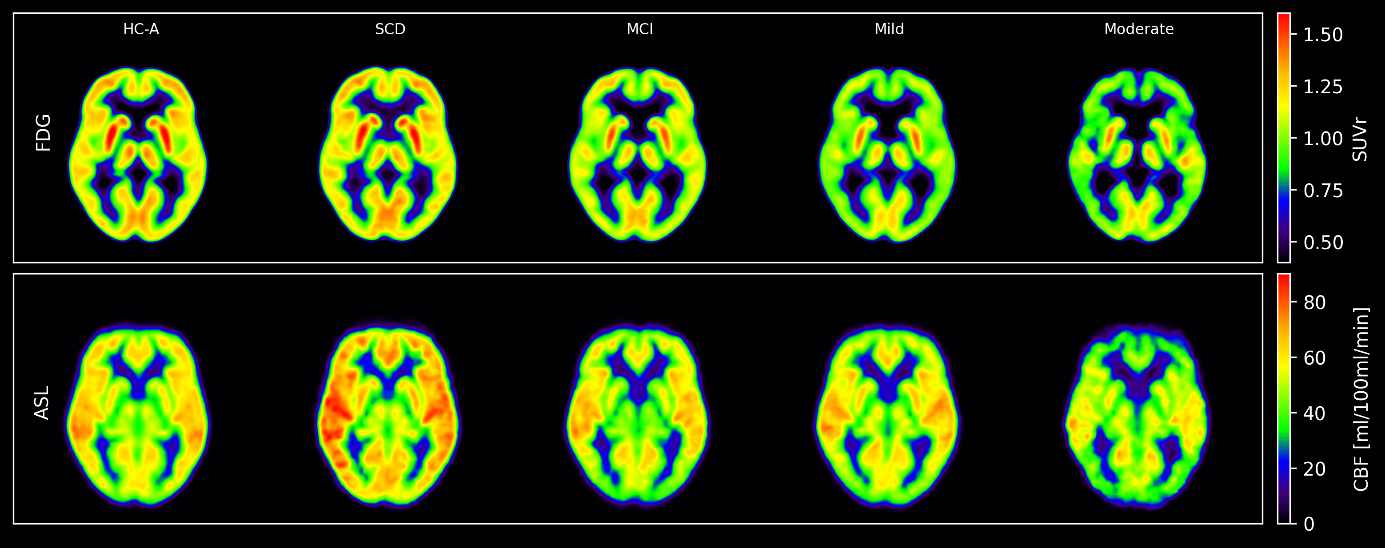
**

**Suppl. Fig. S3.** Mean group images, uninterpretable scan excluded.

**Suppl. Fig. S4** Association of ASL CBF with [^18^F]FDG SUV normalised to cerebral cortex. Scatterplots of hemisphere values in participants with interpretable (black filled circles, n=228 hemispheres) and uninterpretable ASL scans (red hollow circles, n=40 hemispheres) with corresponding regression lines are shown for each region. Crude R^2^ and p-value for diagnostic quality scans are shown. For uninterpretable ASL scans statistically significant associations were observed only for temporal cortex (R^2^=0.144, p=0.016) and parietal cortex (R^2^=0.172, p=0.008).
